# Small-scale habitat heterogeneity and genotype modulate in situ gene expression of the Antarctic bivalve *Aequiyoldia eightsii* in front of a melting glacier

**DOI:** 10.1186/s12862-025-02386-8

**Published:** 2025-07-01

**Authors:** Mariano Martínez, Lars Harms, Doris Abele, Christoph Held

**Affiliations:** 1 Alfred-Wegener-Institut Helmholtz-Zentrum für Polar- und Meeresforschung, Am Handelshafen 12, 27570 Bremerhaven, Germany; 2 Helmholtz Institute for Functional Marine Biodiversity at the University of Oldenburg (HIFMB), Ammerländer Heerstraße 231, 26129 Oldenburg, Germany; 3 Oceanografía y Ecología Marina, Instituto de Ecología y Ciencias Ambientales, Facultad de Ciencias, Universidad de la República, Iguá, Montevideo, 4225, 11400 Uruguay

**Keywords:** Antarctica, Heteroplasmy, Phenotypic plasticity, Protobranchia, Transcriptomics, Single nucleotide polymorphisms

## Abstract

Regulation of gene expression is a pivotal adaptive mechanism of organisms facing environmental variation. We studied the in situ gene expression of the shallow-water bivalve *Aequiyoldia eightsii* in Potter Cove (King George Island, Antarctica) occupying different habitats in front of a melting glacier on a local scale (1 km). The expression of nuclear genes was determined by (1) variation of the nuclear genome itself (nuclear SNPs) and equally strongly by (2) different environmental conditions characterizing the three locations and (3) the composition of the mitochondrial genotype (mitochondrial SNPs). Mitochondrial SNPs divided Antarctic animals into two groups, each composed of organisms featuring mitochondrial homoplasmy and heteroplasmy. We validated our results by contrasting the observed magnitudes of differentially expressed genes (DEGs) with magnitudes expected by stochasticity in randomized group comparisons. Habitat comparison revealed further differences in DEGs at local scale suggesting a high evolutionary adaptive potential to the specific microenvironments. Interestingly, differential expression analysis between mitotypes resulted in a higher number of DEGs than the obtained in the comparison by stations, distinguishing heteroplasmic from homoplasmic organisms. Our results suggest that the mitochondrial genome and possibly heteroplasmy may play a role in the regulation of nuclear gene expression with adaptive implications.

**Supplementary Information:**

The online version contains supplementary material available at 10.1186/s12862-025-02386-8.

## Introduction

In the context of rapid environmental change, phenotypic plasticity has been proposed as a key mechanism for the persistence of organisms in changing environments until genetic adaptation takes place [1]. However, there are not many in situ marine studies looking into how phenotypic plasticity is actually realised, which genetic mechanisms are involved and what the most important determinants are that decide which of the many possible phenotypes actually becomes realized [2]; these questions are mostly addressed through experimental approaches [3].

The model species of this study, *Aequiyoldia eightsii* (Jay, 1839) is a bivalve belonging to the family Sareptidae within the basal group Protobranchia. This species is commonly found in shallow marine waters along the West Antarctic Peninsula (WAP), one of the fastest warming regions on Earth [4]. Occurring in shallow coastal waters, this species is exposed to rapidly changing conditions driven by fast increase of both annual and seasonal air temperature, upper ocean temperature [5, 6], accelerated retreat velocity of land and tidewater glaciers, and progressive melting of the West Antarctic ice sheet. Glacial and sea ice melting, in turn, result in reduced and more strongly fluctuating salinity, enhanced turbidity, and the alteration of nutrient and carbon biogeochemistry in coastal areas [7]. Its frequent occurrence in coastal sedimentary habitats renders this species an excellent model to investigate adaptive responses of Antarctic benthic fauna to the effects of global warming and associated environmental changes. In fact, several studies have evaluated population-specific physiological and behavioral responses to warming and iceberg scouring in this species [8–11]. Clark et al. [9] classified this species as relatively resistant to thermal stress, noting an upper lethal temperature of 25 °C when warmed at a rate of 1 °C h⁻¹. However, Peck et al. [10] showed that the species did not change its upper lethal temperature after 60 days of acclimation to 3.5 °C above the annual average, suggesting poor thermal acclimation ability. In earlier work, Abele et al. [8] tested the effects of warming on routine and basal metabolism, finding a moderate increase in routine metabolism between − 1 °C and + 2 °C, with a more pronounced rise above 2 °C. They also observed that burrowing bouts increased in frequency but became shorter as energy reserves were depleted, and that superoxide dismutase (SOD) activity was inhibited, leading to increased oxidative damage. While distribution records of this nominal species extend to both sides of the Drake Passage in Southern South America and the WAP, recent studies based on mitochondrial and nuclear data suggest cryptic speciation with a pair of two sister species inhabiting South American and Antarctic waters, respectively [12, 13]. For the sake of simplicity, we will be referring to the Antarctic material as *A. eightsii* on the grounds of this material being collected near the *locus typicus* of the original species description (South Shetland Islands). The recent discoveries of the genetic characteristics of this nominal species are not only relevant at inter-continental or population level, but also within the populations down to the level of the individual. In a previous study several highly differentiated mitochondrial genomes coexisting in Antarctic populations but also coexisting in single individuals (heteroplasmy) were reported in a subset of the sampled individuals [13]. The two major haplotypes in the population (h1 and h2; p-dist. 0.008) were mostly observed in homoplasmy (i.e. all mitochondria in one individual being of the same type), but occasionally in combination with a highly divergent haplotype h3 (p-dist. 0.06) in heteroplasmic individuals h1h3 and h2h3. Whether the persistence of distinct mitochondria in the Southern Ocean bivalve *A. eightsii* is indicative of a selective advantage for each of the existing mitotypes is still an open question. Even more intriguing is the prevalence of heteroplasmic individuals in the population that have mitochondria with different mitochondrial genomes coexisting in different tissues in the same individual, since this condition is commonly conceived as unfavourable and affecting mito-nuclear interactions [14, 15].

Mitochondrial heteroplasmy has been reported in over 100 bivalve species related to an evolutionarily stable system known as Doubly Uniparental Inheritance (DUI) [16–18]; which implies females inheriting mitochondria from their mothers (F-type) and males from both paternal lines (F-type and M-type). In males, the F-type is mostly present in somatic tissues whereas the M-type is dominant in the gonads but can also be present in the somatic tissue. Despite its wide distribution across bivalve species and its long evolutionary history, the functional advantages of this heteroplasmic system and its evolutionary significance are still mostly unknown [14]. Recently, Bettinazzi et al. [19, 20] found a significant divergence in sperm performance and partially in energy metabolism strategy between DUI and strict maternal inheritance (SMI) species showing a case of a mitochondrial genotype (mtDNA) responsible for sex-specific (male) functions.

In order to explore these functional and evolutionary aspects in more detail, various molecular approaches can be employed. Transcriptomic sequencing (RNA-seq) has become useful for identifying biological functions or processes as it uncovers patterns that can only emerge from simultaneously studying a large set of genes. Thus, gene expression patterns can reflect biological states or phenotypes responding to specific environmental conditions, as well as the genotype of individuals, allowing unprecedented mechanistic insight into the processes of plasticity and adaptation [21]. However, the normally low proportion of annotated genes in non-model species, and the high number of false positives in differential expression analyses [22] indicates the need for a careful interpretation of the emerging patterns.

In this study, we aimed to understand how the mitochondrial and nuclear genotypic background influences gene transcription patterns in *A. eightsii*, comparing animals from three different small-scale habitats in front of a melting glacier in Potter Cove (WAP) on a local (~ 1 km) scale. We hypothesized that the heteroplasmy or homoplasmy of the mitochondria affects nuclear gene expression within each of the three habitats. Additionally, we assumed that the small-scale habitat variability in Potter Cove causes adaptive modifications of gene transcription in the bivalves, potentially leading to a diversification of genotype-controlled gene expression patterns. To test these hypotheses, we explored gene expression patterns in response to (1) contrasting environmental conditions at local scales (ca. 1 km) considering genetic structuring of the population based on Single Nucleotide Polymorphisms (SNPs), as well as in relation to (2) nuclear and mitochondrial genetic diversity. We validated our results by contrasting the observed magnitudes of differentially expressed genes (DEGs) with magnitudes expected by randomized group comparisons.

## Materials and methods

### Study area

**Fig. 1:**
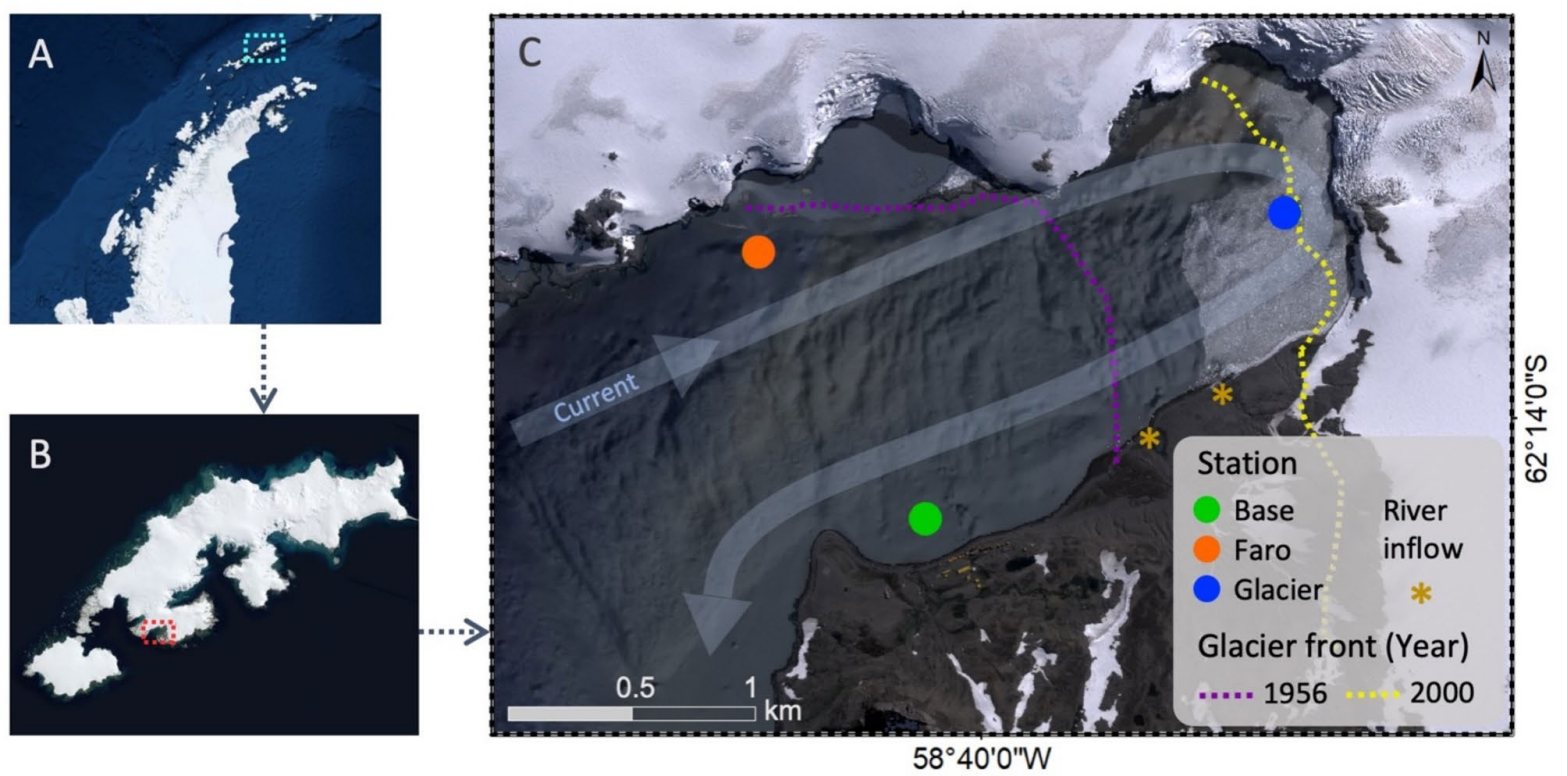
Study area: West Antarctic Peninsula (**A**), King George Island (**B**) and Potter Cove (**C**). Sampled stations Base, Glacier and Faro are indicated with colours. Dashed lines indicate the Glacier front position over time. (modified from Rückamp et al. [30]). The curved arrow shows the predominant direction of the water circulation in the Cove, and orange stars indicate meltwater streams inflow in the Cove. Maps obtained from DigitalGlobe (2013)

Field work was carried out at Potter Cove (PC), a small fjord on the southern coast of King George (South Shetland Islands, Antarctic Peninsula, Fig. 1). Potter Cove is about 4 km long and 2.5 km wide opening into the bigger Maxwell Bay. The northern and the eastern coast are surrounded by the Fourcade Glacier, whereas the southern coast has flat gravel and sandy beaches, intersected by meltwater streams that drain the Warzawa Ice Field and its proglacial lakes [23]. During the summer melt season, the inner cove receives freshwater and ice input directly through the grounding line of Fourcade glacier and from two bigger meltwater streams. A major fraction (approximately 50%) of eroded subglacial and terrigenous sediment particles discharging with the meltwater are deposited within the narrow and relatively shallow parts of the inner cove (maximum depth of 50 m) close to the glacier front [24, 25]. Sedimentation is supported by a prolonged residence time and internal circulation of surface water in the inner cove before the water exits across the internal moraine along the southern coast into Maxwell Bay [26].

The three stations sampled in the present study (Base: 62°14’11"S − 58°40’14"W; Glacier: 62°13’32"S − 58°38’31"W; Faro: 62°13’35"S − 58°40’58"W; Fig. 1) were selected to compare reference sites of contrasting glacial influence within the inner cove, with confirmed presence of *Aequiyoldia eightsii* in the benthic communities [27]. The three stations also represent distinct benthic sub-habitats defined by Jerosch et al. [28] based on cluster analysis of 42 environmental variables. The station “Faro” is situated on the northern coastline and characterized by high bed shear stress and wave action of the inflowing water masses [26] with large grain size and little fine sediment deposition [25]. In contrast, the station “Glacier” receives fine glacial and meltwater stream deposits and is under a strong influence of glacial calving and ice-scouring [29], both leading to frequent resuspension and re-deposition of the fine sediments. The sediment at station “Base” is also composed of fine-grained, muddy material, but has a much higher content of organic matter from decaying macroalgal debris. Furthermore “Base” is situated down-stream of Carlini station which may also explain part of the organic matter enrichment in the area. “Base” has the longest ice-free history (> 50 years), whereas “Glacier” is the most recently ice-free area, uncovered by glacial ice since the early 2000s.

### Sampling

*A. eightsii* collection and environmental characterization of stations in Potter Cove were performed in the austral summer season in January and February 2018. Sampling of bivalves and sediment coring, as well as CTD (conductivity, temperature, and depth)profiling down to the maximum depth of stations were carried out simultaneously at each station. Sampling dates of the three stations were approximately one week apart.

#### Environmental characterization

At each station, salinity, temperature and turbidity profiles were performed with a Sea-Bird CTD (SBE19plusV2, Sea-Bird Electronics, Bellevue, WA, USA). Sediment cores were taken by SCUBA divers using cylindrical Plexiglas corer (height: 50 cm, diameter: 8 cm; 3 replicates per station). The open corers were pushed up to 30 cm into the sediment, sealed with a rubber tap and carefully pulled out with the overlying bottom water to preserve the first layer of the sediment undisturbed. Cores were returned to the laboratory in an upright position for further analysis. In the laboratory, the overlying water was removed and the sediment sliced into 1 cm thick slices. The sediment-water slurry of each slice was mechanically homogenized and separated into two subsamples for grain-size analysis and determination of total inorganic carbon (TIC), total organic carbon (TOC), total nitrogen (TN) and total sulphur (TS). All samples were frozen at − 80 °C for 24 h. Afterwards, the samples were freeze dried for 96 h for transportation.

#### Animal collection

*A. eightsii* were collected in the three stations using a Van Veen grab (n_Base_ = 84, n_Glacier_ = 10, n_Faro_ = 10). Bivalves were transported to the local research facility in insulated containers with water and sediments from their sampling site. At the laboratory, bivalves foot and mantle tissue were dissected on ice under a stereomicroscope after recording individual weight, shell length and width. Bivalves sampled for differential gene expression analysis were dissected within 2 h after collection whereas animals analysed exclusively for Single Nucleotide Polymorphisms (SNPs) were kept alive in aquaria with water and sediment from the Cove at 1 °C (in situ water temperature) and dissected within 20 days after collection. For each individual, foot and mantle tissue samples were conserved separately in RNA later (SIGMA).

### Laboratory and data analysis

#### Sedimentary parameters

For the grain size determination, freeze-dried samples were dry sieved through a 2 mm strainer and weighed in order to separate gravel from the smaller grain size fractions. Afterwards, sediment aggregation < 2 mm was suspended overnight in demineralised water and wet sieved through a 0.063 mm strainer to separate the sand fraction from the silt and clay fraction. The sand fraction was subsequently dried at 40 °C for one week and weighed to the nearest 1 mg. Silt and clay fractions were placed with water in a 5 L-jar and stood for one week for sedimentation. Subsequently, the overlying water was decanted, and the samples stored at − 80 °C for 24 h and freeze dried again for 96 h. Finally, the dry silt-clay-samples were weighed on a fine scale to the nearest 1 mg. With the weights obtained for each grain size fraction, the percentages of gravel and sand, and silt and clay were calculated for each station.

For chemical analyses, samples were pulverized using a sediment mill at the laboratory, and the determination of the total amounts of carbon (TC), nitrogen (TN) and sulphur (TS) was carried out using a gas-phase chromatograph vario EL III (Elementar, Germany). Organic and inorganic carbon (TOC and TIC, respectively) contents were determined by combustion using a Carbon-Sulfur Analyzer CS-2000 (Eltra, Germany). In addition, TOC -sulphur and nitrogen ratios (C: S, C: N) were calculated and used as proxies of sediment oxygenation conditions [31] and organic matter origin (marine vs. terrestrial) [32]. Based on TIC content, the amount of calcium carbonate (CaCO_3_) was calculated according to [23].

**Fig. 2:**
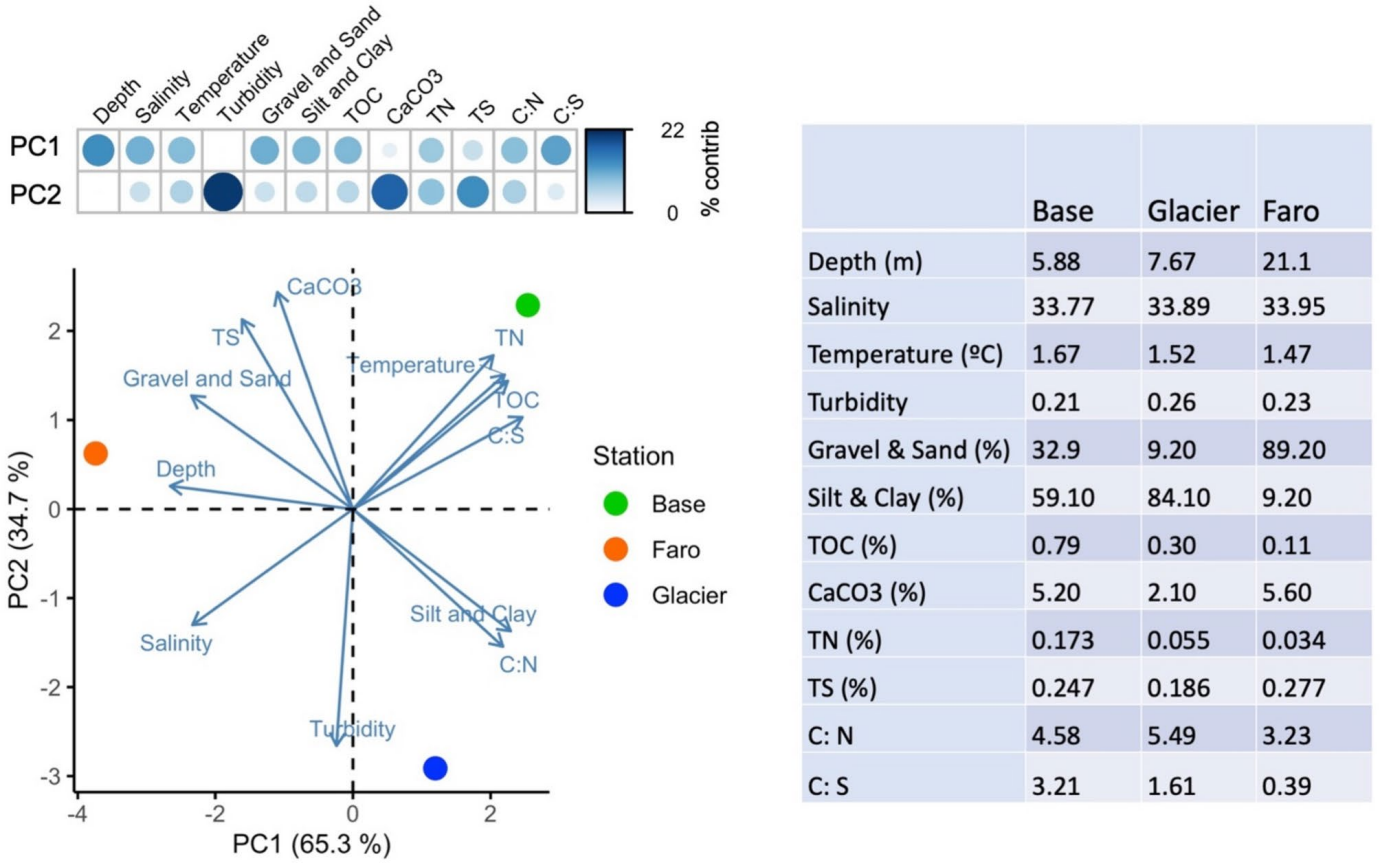
Principal Component Analysis Biplot of stations and environmental variables (left), percentage of contribution of each variable to Principal components (top left), and values of each variable for every station (right). Sediment variables are reported as the average of the 0–2 cm layer values of the three Corer replicates and water variables at the depth closest to the seabed as recorded by CTD (Base: 5.88 m, Glacier: 7.67 m, Faro: 21.1 m)

As *A. eightsii* is normally found within the 2-cm sediment depth [33], results of the sedimentary parameters for each station are presented as the average of the first two layers of sediment (0–1 cm and 1–2 cm). For a better visualization of the environmental characteristics of the stations, a Principal Component Analysis Biplot was performed (Fig. 2).

#### RNA extraction, library Preparation and de Novo assembly

Differential gene expression and Single Nucleotide Polymorphisms (SNPs) on RNA-seq data were performed using an assembly created from 70 libraries (one library per individual) of *A. eightsii* from South America (Punta Arenas, Chile) and Antarctica (Potter Cove) by Martinez et al. [13]. The samples analyzed in the present study were used for the assembly performed in the previous study. Thus, in the present study, 31 libraries from Potter Cove were included in the transcriptomic analysis (n_Base_ = 21, n_Glacier_ = 5, n_Faro_ = 5), all of them analysed for nuclear and mitochondrial SNPs on RNA-seq data, and a subset of 15 individual samples (corresponding to organisms dissected immediately after collection, i.e., representing in situ conditions) were analysed for differential gene expression (n_Base_ = 5, n_Glacier_ = 5, n_Faro_ = 5). RNA was extracted from mantle tissue (5–30 mg) by homogenizing the samples in Trizol reagent (SIGMA) using a Precellys homogenizer (Precellys24, Bertin Technologies, France). Total RNA was isolated from each sample using the Direct-zol™ RNA MiniPrep Kit (ZYMO Research Corp., USA) according to the manufacturer’s instructions. Libraries were prepared using the Illumina TruSeq Stranded mRNA Sample Preparation Kit starting from 1 µg of total RNA following the protocol provided by the kit. During libraries preparation, each sample was tagged through adapters ligation. Each cDNA library was dilued to 0.8 nM, and subsequently all the libraries were pooled and cleaned using magnetic beads to remove the remaining primer content. Final cDNA concentration was measured in the LabChip GX Touch (PerkinElmer, USA). The pool of samples was sequenced on an Illumina NextSeq 500 sequencer using the NextSeq High Output Kit v2 (150 cycles) with a paired-end protocol.

Raw reads were quality controlled by FastQC v. 0.11.7 (Babraham Institute, Cambridge, UK), cleaned and *de novo* assembled using the Trinity genome-independent transcriptome assembler v2.8.4 [34]. Duplicate sequences were removed using dedupe.sh from the BBmap toolkit [35] with the following parameters: minidentity = 98, arc = t, am = t) and ac = t.For details on *de novo* assembly parameters see Martinez et al. [13].

#### Single nucleotide polymorphisms analysis (SNP)

The SNP analysis involved the alignment of quality-filtered paired-end reads to the *de novo* transcriptome using bowtie2 v2.3.4.1 [36]. Alignments in SAM format (Sequence Alignment Map) were compressed and indexed with SAMtools v1.8 [37]. Genotype likelihoods were computed using mpileup from SAMtools and variant calling was performed with the BCFtools. In a first filtering step, variants with a Phred quality score below 30 were excluded. A total of 927,305 SNPs in 31 individuals were retained. Subsequently, an iterative filtering strategy between *loci* and individuals with a progressive increase of cut-off values [38] was performed using VCFtools v0.1.16 [39]. A first filtering for *loci* quality was performed keeping variants successfully genotyped in 50% of individuals (max-missing 0.5) and with a minor allele count (MAC) of 3. Afterwards, individuals with more than 27% of missing data were removed from the analysis. A second filtering for *loci* quality consisted in a max-missing of 0.95, a Minor Allele Frequency (MAF) of 0.05 and minimum number of reads (minDP) of 10. Additionally, variants with more than two alleles were discarded (max-alleles 2). After filtering, 120,404 SNPs in 27 individuals (= libraries) were kept. The retained variants were retrospectively identified as nuclear and mitochondrial SNPs through blasting the sequences containing SNPs against a mitochondrial database obtained from the UniProt Swiss-Prot database. Nuclear SNPs comprised all SNPs in sequences with no hits in the mitochondrial database. Mitochondrial SNPs included in the analysis were those contained in a single long contig (ca. 18 kbp), representing most of the mitochondrial genome (annotated using MITOS following [40], see Martinez et al. [13]). Thus, the analyses were performed with a total of 145 and 116,123 mitochondrial and nuclear SNPs, respectively.

To assess genetic differentiation between stations, Analysis of Molecular Variance (AMOVA), Principal Component Analyses (PCA), and pairwise G_ST_’ estimations were carried out separately for nuclear and mitochondrial SNPs with all the individuals that remained after the filtering. AMOVA of mitochondrial and nuclear SNPs was conducted considering two strata, station (Base, Glacier and Faro) and individuals, and significance was tested by randomly permutating (*n* = 1,000) the sample matrices following Excoffier et al. [41] (Table 1). In both PCAs visualizing mitochondrial and nuclear SNPs, every individual was labeled based on the sampling station and mitochondrial mitotype. Mitotype of individuals were determined in Martinez et al. [13], through the amplification of a 714 bp *Cytochrome c oxidase subunit I* fragment using haplotype-specific primers. In the mentioned study, bivalves were grouped in six different genetic clusters: animals carrying only one haplotype (h1, h2, h3, h4), named h1h1, h2h2, h3h3 and h4h4 respectively, and heteroplasmic animals for haplotype h1 or h2 together with the highly divergent haplotype h3 (p-dist. 0.06 respect to h1 and h2), called h1h3 and h2h3, respectively. The current study analyzed a subset of animals from Potter Cove only carrying mitotypes h1h1, h1h2, h1h3 and h2h3, since h3h3 individuals were not represented in the SNPs analysis and the h4h4 mitotype is exclusive to South American organisms.

All the analyses were performed in R v3.6 [42] using the packages vcfR [43], adegenet [44], poppr v2.8.3 [45], ade4 [46] and pegas [47].

#### Differential gene expression and gene ontology analyses

The differential gene expression analysis (DEA) involved the alignment of the short reads of each sample (*n* = 15) separately against the de novo reference transcriptome using Bowtie2 v 3.3.4.1 [36]. The DEA implied pairwise comparisons between clusters of individuals grouped by stations (n_Base_ = 5, n_Glacier_ = 5, n_Faro_ = 5), but also grouped based on their mitotype (n_h1h1_ = 2, n_h2h2_ = 7, n_h1h3_ = 3, n_h2h3_ = 3) and nuclear type (n_NucA_ = 10, n_NucB_ = 5, see Sect. 3.3), using the same set of 15 individual samples; called from here onwards the ‘original grouping’. To minimize the complexity of data interpretation, the differential gene expression analysis was restricted to pairwise comparisons. Potential interaction effects between experimental factors were not assessed. To assess the presence of random or stochastic effects on gene expression, if any, we performed DEAs (*n* = 1,000) using randomized groups of the same 15 individual samples following the same numerical conformation of the original grouping (i.e. stations: *n* = 5, *n* = 5, *n* = 5; mitotype: *n* = 2, *n* = 7, *n* = 3, *n* = 3; nuclear type: *n* = 10, *n* = 5). Gamma distribution functions were selected based on Akaike information criterion (AIC) and fitted to each of the three data sets of differential expressed genes (DEGs) generated by randomized grouping, and the quantiles 0.95 (Q_0.95_) and confidence intervals for the Q_0.95_ were calculated following Kulkarni and Powar [48] using the R package EnvStats. With this, we tested if the observed values of DEGs generated by the original groupings were above the Q_0.95_ of the gamma distribution of DEGs values obtained from DEAs (*n* = 1,000) with randomized groups of individuals. For every DEA, relative abundances were estimated by RSEM v1.2.26 [49] and DEG were assessed using a test based on the negative binomial distribution as integrated in the Bioconductor R package DESeq2 [50], with the significance threshold set to *p* ≤ 0.01 and a fold change of at least 2. The p-value was corrected for multiple testing using an FDR correction (Benjamini-Hochberg procedure) applied by default in the package DESeq2.The tools were executed using the Trinity package v2.8.4. The annotation of DEGs was performed using the Trinotate functional annotation suite v3.1.1 [34] including a homology search against the UniProt Swiss-Prot database and assigning Gene Ontology (GO) terms to annotated transcripts. GO enrichment analyses were carried out using GOseq [51] with a p-value threshold of 0.05 by combining the results of DEGs between each cluster and its corresponding GO annotation, and using the full list of DEG with GO terms as the background.

## Results

### Environmental conditions

The environmental characteristics captured in the water column and sedimentary parameters indicate that the stations Base and Glacier are mostly under the influence of sediment deposition from subglacial erosion and meltwater streams whereas station Faro is receiving strong currents of inflowing water from the outer Potter Cove and Bransfield Strait. The Principal Component Analysis Biplot (Fig. 2) separated the stations Base and Glacier from Faro along the Principal Component axis 1 (PC1), while the axis PC2 mostly discriminated Base and Faro from Glacier. PC1 discriminated stations Base and Glacier from Faro based on higher bottom water temperature, and percentage of total organic carbon (TOC) and nitrogen (TN) in surface sediments at Base, and higher percentage of silt and clay at Glacier; whereas Faro is the deepest station with relatively higher bottom water salinity, and a bottom cover of mainly gravel and sand. Bottom water turbidity, calcium carbonate (CaCO_3_) and total sulphur (TS) were the main factors represented by PC2 with the highest turbidity at Glacier and higher CaCO_3_ and TS percentages at Base and Faro compared to Glacier. Bottom water parameters followed the general trend of the water column profiles among stations although with less pronounced differences (Supporting Information Figure S1). The ratio between TOC and TS (C: S) increased from Faro to Glacier and to Base along PC1 with values suggesting, according to Togunwa and Abdullah [31], near anoxic conditions in Faro and suboxic conditions in Base and Glacier. Instead, the ratio between TOC and TN (C: N) showed slight differences between stations with values within the range characteristic for marine ecosystem components (such as macroalgal debris, phytoplankton deposits and benthic microalgal mats).

Although *Aequiyoldia eightsii* density at each station was not explicitly determined, the higher sampling effort required at Faro for the collection of a lower individual number in comparison to Base and Glacier suggests a comparatively poorer habitat suitability with soft sediment confined to small patches within the rocky bottom macroalgal belt. Both mean and maximum shell length of our samples were higher at Base (2.28/3.3 cm) than Glacier (2.18/2.9 cm) and Faro (2.22/2.5 cm).

### Genetic structure

Analysis of Molecular Variance (AMOVA) based on mitochondrial SNPs did not show genetic structuring between stations, whereas AMOVA based on nuclear SNPs showed a slight but significant spatial genetic differentiation. In both cases most of the variance (97.84 and 96.53%, respectively) was among individuals and only 2.16% and 3.47% between stations, respectively (Table 1).

**Table 1:** Coefficients of the analysis of molecular variance (AMOVA) of mitochondrial (Mit) and nuclear (Nuc) SNPs between stations

Source of variation	df		Sum of squares		Percentage of variation		*p* value	
	Mit.	Nuc.	Mit.	Nuc.	Mit	Nuc.	Mit.	Nuc.
Between stations	2	2	0.41	0.31	2.16	3.47	0.31	0.001
Within individuals	24	24	4.23	2.99	97.84	96.53		

df: degree of freedom

**Fig. 3:**
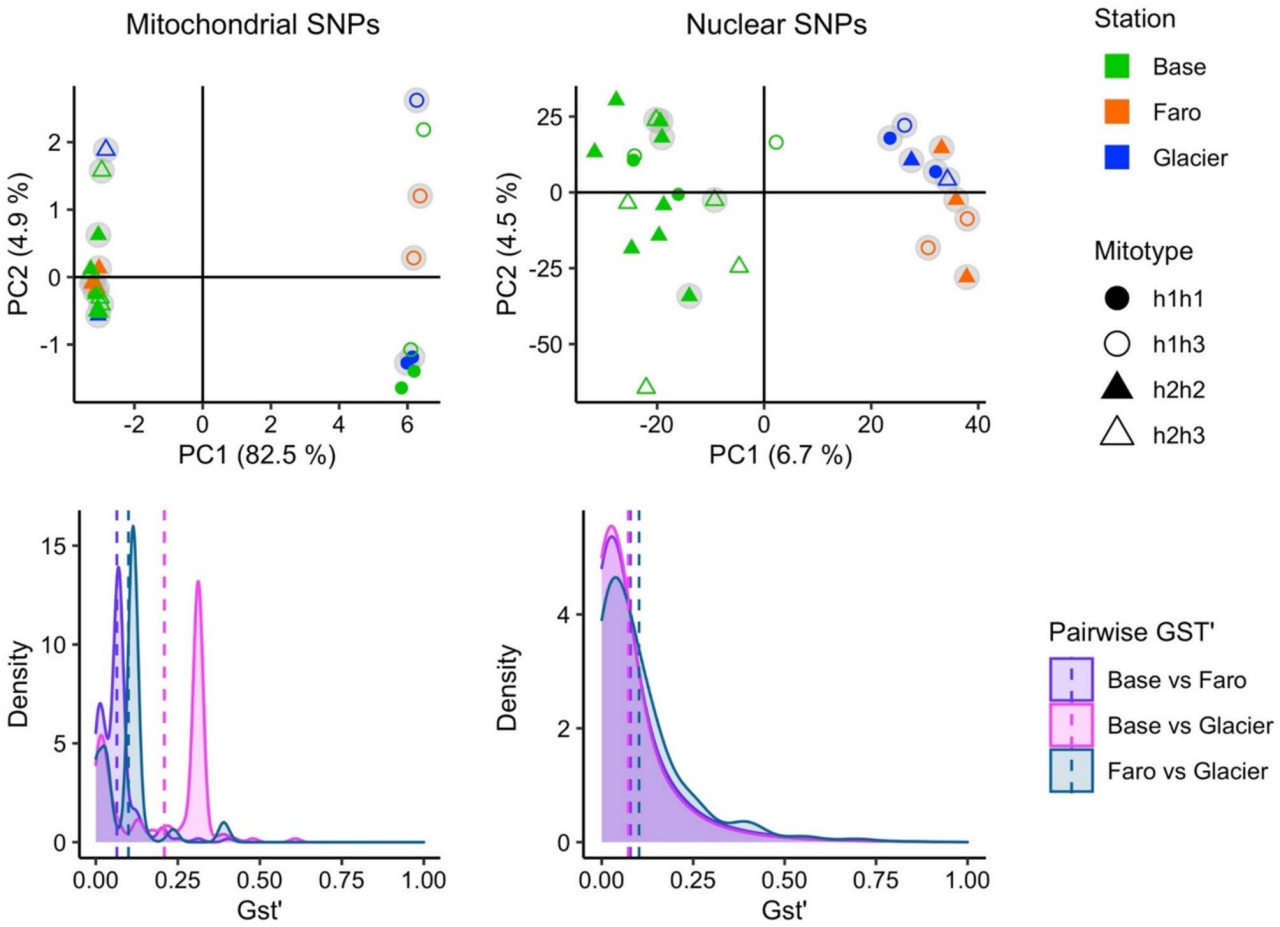
Principal component analysis based on mitochondrial and nuclear SNPs of bivalves *Aequiyoldia eightsii* collected in Base, Faro and Glacier stations in Potter Cove (panels above). Sampling stations are indicated with colours, mitotypes with shapes and grey circles highlight individuals that were included in the differential gene expression analysis. In the panels below, the density distributions (Kernel density estimate) of G_ST_’ values in pairwise comparison of animals from the three stations based on mitochondrial and nuclear SNPs are shown. Dashed lines represent the mean G_ST_’ of the correspondingly coloured distribution

The AMOVA results are in accordance with the Principal Component Analysis of mitochondrial and nuclear SNP data (Fig. 3). Previously [13], it has been shown that mitochondrial SNPs in *A. eightsii* from Potter Cove fall into two main groups characterized by haplotypes h1 or h2, either in homoplasmic or in heteroplasmic condition with haplotype h3 (Fig. 3, left: h1h3, h2h3). This distinction of two major mitotype groups (**h1**h1or **h1**h3) vs. (**h2**h2 or **h2**h3) dominates the separation along PC1 (82.5%) but no accompanying spatial pattern in Potter Cove is discernible. With the exception of h1h1 and h2h3, which we did not sample at station Faro, all mitotypes were found at every station sampled.

In contrast, the nuclear SNPs divided the animals into groups that are not congruent with the major mitotypes (Fig. 3, right). Instead, a weak but significant spatial pattern emerged, discriminating the animals of station Base from a group comprising animals from Faro and Glacier, but the variance explained by each component was low (PC1: 6.7%, PC2: 4.5%) especially when compared to the variance explained in the PCA analysis conducted with mitochondrial SNPs. Pairwise G_ST_’ comparisons (Fig. 3, bottom half) also indicated a low level of genetic structuring between stations, with the highest mitochondrial genetic difference between Base and Glacier stations (bimodal distribution), and rather similar G_ST_’ density distributions at every nuclear pairwise comparison between stations.

Both groupings, the one based on mitochondrial (mitotypes) and the one based on nuclear SNPs (nuclear type: NucA: individuals from Base, NucB: individuals from Faro and Glacier) were considered for the gene expression analysis. While mitotypes can be considered mitochondrial genotypes, as they were defined based on mitochondrial SNPs across the entire mitochondrial genome and Sanger sequencing, nuclear types are more appropriately described as groupings of organisms based solely on SNP similarities, without sufficient evidence to define them as genotypes. The absence of a strong genetic structure between stations, as demonstrated by the previous results, allows for the discrimination between environmental (station) effects on gene expression and those driven by the genetic background.

### Gene expression

#### Differential gene expression

**Fig. 4:**
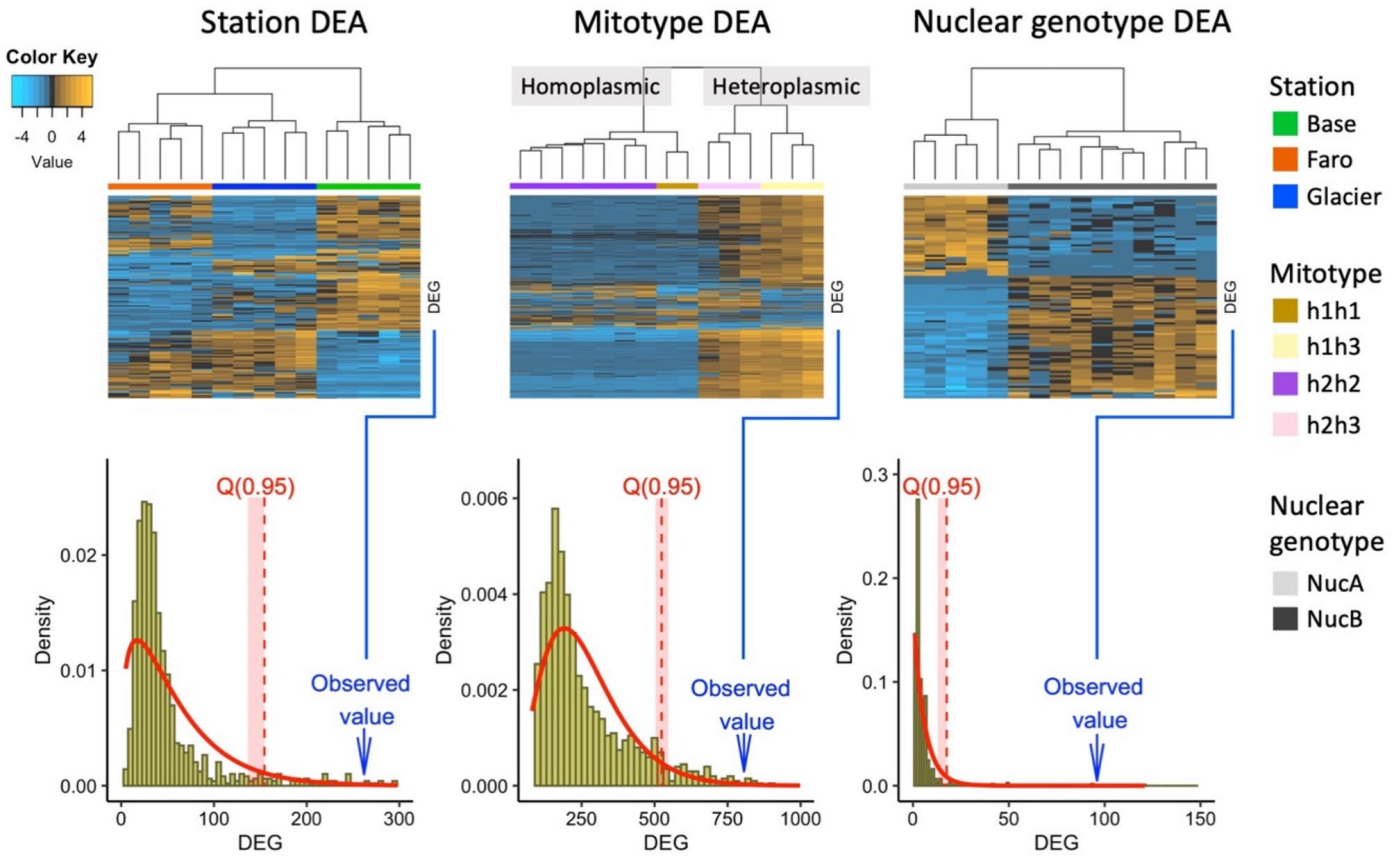
Above: heatmaps showing the differentially expressed genes (DEG) in the three differential expression analyses (DEA) considering individuals grouped by station, mitotype and nuclear type. For every heatmap, a cluster analysis showing similarities between groups is included on top. DEGs are displayed in rows and counts per sample are displayed in columns. Genes included were significant (*p* < 0.001) in at least one pairwise comparison. A colour code indicates up-regulated and down-regulated expression levels (top left). Below: density distributions of DEG values obtained from DEAs (*n* = 1000) with randomized groups of individuals. The red curve corresponds to the adjusted gamma distribution and the dashed red line and area underlying indicate the Quantile 0.95 (Q_0.95_) and Confidence interval of the Q_0.95_. In every case, the observed DEG value was above the Q_0.95_

Differential gene expression analysis (DEA) considering clustering of individuals by station or by genotypes (mitotype and nuclear type) resulted in different numbers of differentially expressed genes (DEGs), all well above the number of DEGs expected by random individual grouping; i.e., the observed numbers of DEGs were above the quantile 0.95 (Q_0.95_) of the gamma distribution of DEG values obtained from DEAs (*n* = 1000) with randomized groups of individuals (Fig. 4).

The pairwise comparison between animals grouped by stations resulted in 262 observed DEGs, with the Q_0.95_ of the adjusted gamma distribution of 154.5 expected by stochasticity alone (Fig. 4 bottom). The comparison between animals grouped by mitotype and nuclear type resulted in 806 and 96 DEG, and Q_0.95_ of 524.1 and 17.6, respectively. The DEA based on stations showed Glacier and Faro clustered together with a relatively low number of genes differentially expressed between them (37 DEGs), while more than three times as many genes were significantly up- or downregulated between station Base and either of the two others (139 and 121 DEGs, respectively). The highest number of DEGs in comparisons based on mitotype groups was observed between homoplasmic and heteroplasmic mitochondrial clusters (176–600 DEGs), whereas the lowest DEG numbers resulted in the pairwise comparison of h1h1 vs. h2h2 (18 DEGs) and h1h3 vs. h2h3 (41 DEGs). Thus, clustering based on patterns of gene expression clearly separates bivalves with homoplasmic mitochondrial genome from heteroplasmic individuals.

#### Annotation and GO enrichment analysis

**Fig. 5:**
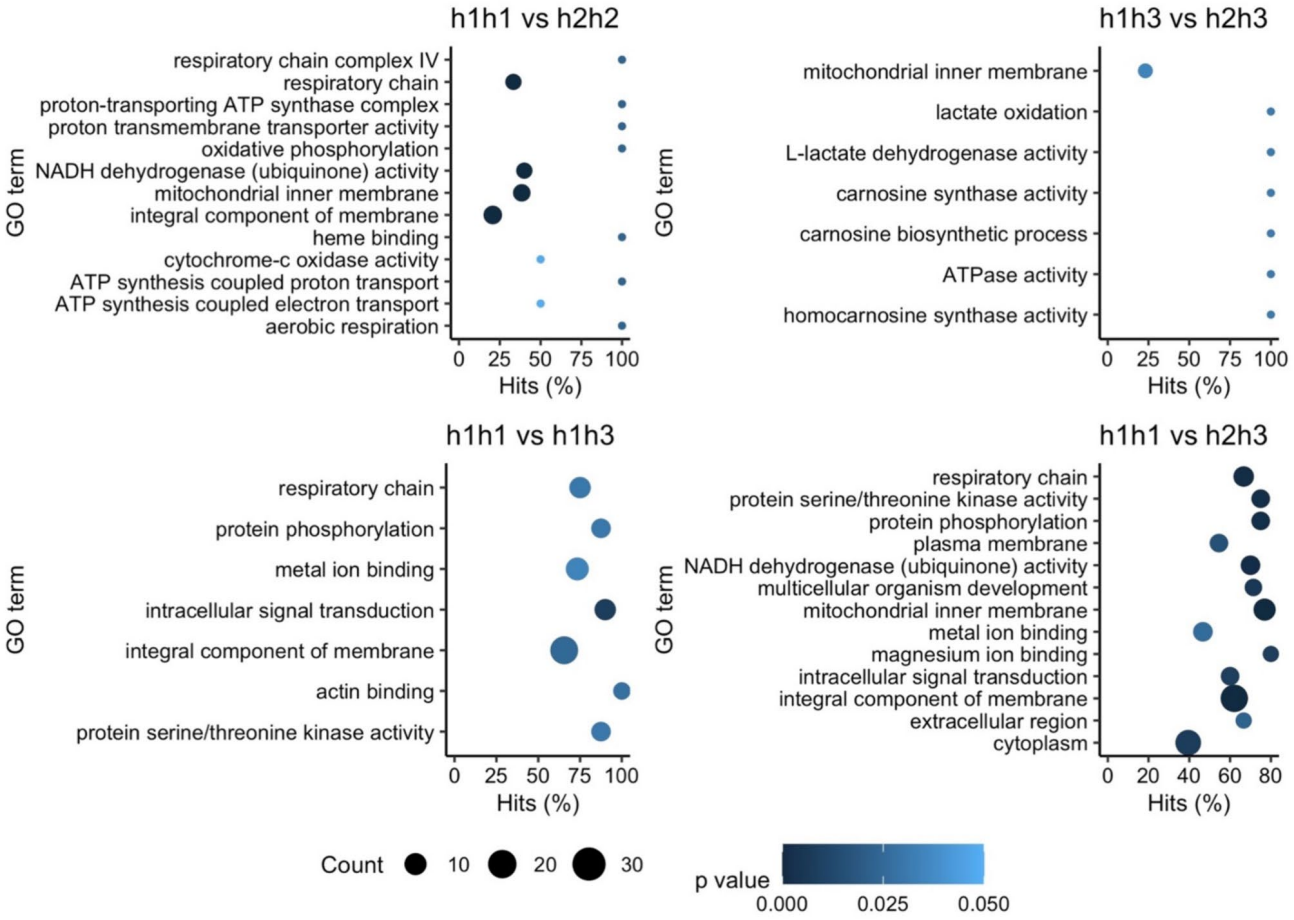
Enriched GO term categories between mitotypes. In the x axis it is shown the percentage of DEGs found in a certain category over the total number of genes in the category (Hits). Size code indicates the number of DEGs in the GO term category and a gradient of colour indicates the p value of the over represented category. For a better visualization, only the 13 most significant GO terms are shown (higher count and p value), as only the comparison h1h1 vs. h2h3 involved a higher number (22 GO terms)

Out of the total number of DEGs obtained in the DEA grouped by station, mitotype and nuclear type, 15.1%, 13.4% and 13.3% could be functionally annotated against the UniProt Swiss-Prot database. Only in the DEA based on mitotype, the GO enrichment analysis resulted in gene categories enriched over a background gene expression. The enriched categories for each mitotype comparison are shown in Fig. 5 and Supporting Information Table S2. Also, the Supporting information Table S3 presents examples of differentially expressed genes associated with the most significant GO terms (the top three for each comparison between mitotypes). In agreement with the DEA, the comparisons between individuals characterized by mitotypes h1h1 vs. h2h2 and h1h3 vs. h2h3 resulted in lower numbers of DEG per enriched GO term category (counts), while every comparison between homoplasmic and heteroplasmic individuals implied a significantly higher number of DEG per enriched category, with the exception of the h2h2 vs. h1h3 comparison which did not result in any enriched category (Fig. 5). The comparison between the homoplasmic groups (h1h1 vs. h2h2) resulted almost exclusively in the enrichment of categories associated with mitochondrial respiration, i.e. energetic functions. However, in comparisons between heteroplasmic groups (h1h3 vs. h2h3) as well as between homoplasmic vs. heteroplasmic individuals, the pattern is less clear. A great diversity of categories involving cytosolic functions were enriched, some of which also involving mitochondrial respiratory functions.

In Supporting Information Table S4 we show a list of Biological Processes related to each GO term obtained from the DEA based on stations and nuclear type which lacks statistical support but summarizes possible differentially activated functions between groups. In these comparisons, genes associated with mitochondrial respiration do not predominate in the same manner as found for the mitotype comparisons. GO terms related to biological processes such us `lactate oxidation´ and `response to hypoxia´ were associated to genes differentially expressed in the DEA by stations in the comparison Base vs. Faro and Glacier vs. Faro, respectively. These results seem plausible in the context of oxygenation in the upper sediment horizons, the burrowing zone of *A. eightsii*, but the lack of statistical significance precludes a meaningful analysis based on small-scale spatial or nuclear type at this point.

## Discussion

The Antarctic shallow-water marine bivalve *Aequiyoldia eightsii* has a strikingly differentiated gene expression pattern under natural conditions in Potter Cove (King George Island), a pattern that is correlated with at least three independent underlying causes: microclimatic differences down to a kilometer scale (stations), mitotype (especially admixture of a second mitochondrial genotype per individual) and nuclear type. All three potential determinants of in situ differences in gene expression proved significant if viewed in isolation, even after correcting for spurious correlations due to stochastic effects.

### Gene expression patterns

#### Microhabitat influence

Physiological responses of organisms are dependent on their perception of the environment. In the attempt to understand organism-environment interactions, we often superimpose our own human perceptions on other species and environment. This ultimately leads to a bias towards analysis on ‘human scales’ (spatial and temporal) and the overlooking of mechanisms underlying adaptation of species or populations to their closest surrounding environment. Consequently, there is vast empirical evidence showing regional or large scale-environmental heterogeneity promoting plastic responses in widely distributed species [52–54], but studies showing in situ responses of species to small-scale habitat heterogeneity are very scarce in the literature [55, 56].

The environmental characterization of the three sampled stations reflected the small-scale habitat variability previously reported in Potter Cove [25, 28], which is shaped by a directional current pattern with characteristic input signatures from different terrigenous sources as well as the dynamic melting of the KGI ice sheet and the recession of Fourcade glacier grounding line [23]. The influence of spatial gradients in Potter Cove on biological communities (composition, functional traits) has been previously addressed [27, 57]; however, there are no antecedents of studies evaluating in situ gene expression responses for organisms in this area, and in other marine pro-glacial habitats, on such a fine spatial scale. Our spatial differential gene expression analysis revealed that organisms at each station express a distinct and characteristic set of mitochondrial and nuclear-encoded genes regardless their genetic background (mitotype and nuclear type) possibly enabling plastic responses to specific microenvironmental niches. The ability of *A. eightsii* to precisely modulate gene transcription according to its intimate physico-chemical environment might confer resilience to the current rapid environmental change in many areas of the Antarctic peninsula.

#### Genotype influence (nuclear and mitochondrial)

Mitochondrial and nuclear genotypes both have their own influence on gene expression independently from one another. This is remarkable as it implies that the composition of the mitochondrial genotype is correlated with the expression of genes that are for the most part located in the nuclear genome. The mechanism how the presence of a certain mitochondrial genome in the cell might modulate the transcription of genes located in the cell nucleus is unclear in bivalve species, but Pozzi et al. [58] have detected the presence of small mitochondrial highly-transcribed RNAs (smithRNAs) in the bivalve *Ruditapes philippinarum*, suggesting RNA interference as a mechanistic explanation for nuclear gene expression regulation by mtDNA.

The most striking results regarding the relation between genetic background and gene expression emerged from transcriptional differentiation based on mitotypes. Interestingly, whereas SNP data support a structure dominated by the possession of mitochondrial haplotype h1 or h2: [**h1h1** + **h1**h3] vs. [**h2h2** + **h2**h3], gene expression patterns were similar among all homoplasmic individuals (with either h1 or h2 mitotype) but were highly distinct from all heteroplasmic individuals (h1h3 and h2h3). The admixture of the mitochondrial h3 haplotype in addition to the major mitotypes h1 and h2, even in low dosage, might lead to different gene expression patterns including genes encoded in the nuclear genome (Fig. 4). On the other hand, the fact that organisms were not sexed and samples corresponded exclusively to somatic tissues does not allow us to confirm that heteroplasmy in this species is linked to DUI, although it seems highly likely given the widespread occurrence of this phenomenon across different bivalve subclasses [16]. As heteroplasmy in DUI species is generally restricted to males, the observed differences in gene expression between homoplasmic and heteroplasmic organisms could be the result of the so-called sex-biased genes. However, it is known that sex-biased gene expression tends to be highest in the gonads and low in somatic tissues [59]. Therefore, while it is possible that part of the differentially expressed genes observed between homoplasmic and heteroplasmic organisms in this study is due to sex-biased gene expression, the observed differences also between homoplasmic mitotypes (h1h1 vs. h2h2, with major functional implications, see Sect. *4.2*), and the usually low incidence of sex-biased genes in somatic tissues argue in favour of an important contribution mtDNA and heteroplasmy in itself to the modulation of gene expression in *Aequiyoldia*. Breton et al. [60] found in two freshwater mussel species with DUI of mtDNA (*Utterbackia peninsularis* and *Venustaconcha ellipsiformi*) that F and M mitochondrial genomes are co-expressed in somatic tissues. This finding could suggest that, in case *Aequiyoldia* presents DUI, the differential expression between homoplasmic and heteroplasmic bivalves would be indeed due to the co-expression of F- and M- mitochondrial types in somatic tissues of heteroplasmic organisms vs. the solitary expression of F-type in homoplasmic organisms.

#### Randomness and uncontrolled factors quota

Genes are considered as differentially expressed when they simultaneously satisfy a *p value* and a fold change criterion. No matter how small the number of differentially expressed genes resulting from an analysis might be, their statistical significance is normally considered sufficient to support the biological interpretations emerging from them, or at least to refute the ‘no effects’ null hypothesis. Our analysis in *A. eightsii*, however, showed that even applying rather strict *p* and fold change criteria and involving comparisons lacking biological/ecological meaning (randomized groups), the probability of a differential gene expression analyses (DEA) yielding null results (i.e. zero DEG) in natural populations is virtually zero.

The reason behind the numbers of DEGs resulting from comparison with randomized groups, which in some cases were even higher than the results obtained with the original grouping (i.e. by station, mitotype and nuclear type) has several explanations. Even though DESeq2 is a conservative method with a high number of false negatives and a low number of false positives compared to other methods [22], it is reasonable to expect that among the total number of assembled transcripts, some transcripts appear to be significantly over- or under-expressed merely by chance. The magnitude of this phenomenon likely increases as sample number per group decreases, as it was the case in our study. Additionally, and especially in those cases where the number of DEGs obtained from comparisons with randomized groups resulted in numbers similar or higher than those obtained with the original groupings, this phenomenon is likely to be connected to the plethora of non-controlled factors/ conditions (e.g. sex, age, presence of parasites, etc.) triggering uniform gene expression responses across organisms sharing or experiencing them. This issue is applicable to any biological approach, but transcriptomic profiling makes it visible as it provides a broad account of cellular processes responding simultaneously to a wide range of stimuli. Another explanation could be linked to the increasing evidence of open pangenomas in bivalve species. These pangenomes are marked by significant gene presence/absence variation, with certain genes present in only a subset of individuals within natural populations [61]. The presence of such gene variation could help to explain the extreme fold changes observed in differentially expressed genes (DEGs), particularly when comparing individuals from the same species or population. Therefore, it is not unexpected to observe DEGs in random comparisons between individuals of the same population, as variations in gene presence/absence can significantly influence gene expression patterns. The possibility of interference of other factors to the observed responses as well as the presumable stochastic component in the DEAs is a warning sign with respect to analyzing DEGs individually, and emphasizes the need for statistical post analysis considering DEG as a whole, such as enrichment analyses.

### Functional analysis

The enrichment of nuclear GO terms based on differences in mtDNA (mitotypes), highlights the tight coordination between nuclear and mitochondrial genomes. In model organisms, sequence variation within mtDNA affects patterns of gene expression on the same nuclear background across a wide variety of nuclear genes in mice [62] and key mitochondrial protein-coding genes in *Drosophila* [63]. In our study, mitochondrial functions were also among the most represented in the enrichment analysis, indicating that intra-population mtDNA might imply a differential transcriptional regulation of mitochondrial and nuclear-encoded mitochondrial complex subunits among mitotypes, in support of adequate mitochondrial functioning. Remarkably, in most of the cases the comparisons between homoplasmic and heteroplasmic individuals implied the highest number of DEGs per enriched category (Fig. 5). This suggests that not only the mitochondrial genotype itself (whether homoplasmic or heteroplasmic) significantly influences the expression of nuclear and mitochondrial genes; rather, the heteroplasmic condition of the mitochondria appears to have an even more pronounced effect on gene expression. The influence of mtDNA variations in the phenotype of bivalve species with DUI of the mitochondria was recently reported and further studied [19, 20, 64]. These studies found a reorganization of M-type mitochondrial oxidative phosphorylation including changes in the activity of key metabolic enzymes, and a decrease in sperm speed and altered trajectories. The authors hypothesize that these energetic adaptations of male mitochondria might contribute to avoid degradation during fertilization and thus play a key role in their own selection and transmission throughout generations. The maintenance of two types of mitochondria, with among other things different bioenergetic yields, could also have an eco-evolutionary significance. The differential transcriptional regulation based on differences in mtDNA (mitotype) could afford this species a higher adaptive potential to cope with spatial or temporal variability.

The observed differences in gene expression/mitochondrial functions could also be attributed to variations in mitochondrial densities between organisms/mitotypes. This potential effect cannot be discerned from gene expression per se using our approach. However, considering that bivalves can adjust mitochondrial density in their tissues as part of their adaptive strategies to changing environmental conditions, this ability further supports our hypothesis regarding their remarkable mitochondrial versatility. Marine shallow-water sedimentary environments are in no way homogenous and instead present animals with an enormous variety of redox conditions in space and time [65]. Cohorts of animals differing in mitochondrial respiratory efficiency, manifested for example through changes in gene expression or adjustments in mitochondrial densities, might even confer population-wide plasticity of metabolic function that enables the animals to thrive in different redox environments of the upper sediment horizon. For animals of very reduced horizontal mobility in the adult form as *A. eightsii*, this may be a way to ensure better habitat exploitation and in so doing contribute to the resilience of the fjordic population. The ability of *A. eightsii* to respond to redox conditions was also suggested (although without statistical post-analysis, see Sect. 4.1.2) by the results from DEA based on stations (GO:0019516: lactate oxidation, GO:0001666: response to hypoxia); and recently reported by Martinez et al. [66] in which we found the antarctic *Aequiyoldia* responding to experimental conditions of hypoxia through a metabolic rate depression strategy mediated by the expression of the Alternative oxidase enzyme.

## Conclusions

The conspicuous genetic diversity of *A. eightsii* in the Southern Ocean and in particular the existence of two mitochondrial haplotypes in heteroplasmy, in combination with the reported small-scale habitat and genotype-based phenotypic diversification, denote considerable adaptive potential, not normally expected in Antarctic species [10]. This genomic condition might serve to add another layer of flexibility to react to changes in the environment and might turn out be instrumental in the face of the ongoing rapid environmental change in Antarctic fjords. Ultimately, it may turn *A. eightsii* into a winner of environmental change [67], expanding into habitats still dominated by more susceptible filter feeders many of which are on the retreat already [68].

## Electronic supplementary material

Below is the link to the electronic supplementary material.

Supplementary Material 1

## Data Availability

Raw Illumina reads were deposited in the European nucleotide Archive database (EMBL-EBI) with the accessions ERR4265443 and ERR4276392 – ERR4276460 under the study accession ‘Mitochondrial heteroplasmy as a systematic bias in molecular species delimitation and barcoding’ (ERP122389).
