# Supplementary material for: Small-scale habitat heterogeneity and genotype modulate in situ gene expression of the Antarctic bivalve Aequiyoldia eightsii in front of a melting glacier
Source: BMC Ecol Evol. 2025 Jul 1;25:64. doi: 10.1186/s12862-025-02386-8 (PMC12211206; doi:10.1186/s12862-025-02386-8)
Supplement: Supplementary file 1 — Supplementary Material 1 [file 12862_2025_2386_MOESM1_ESM.docx]

**Supporting Information for:**

Small-scale habitat heterogeneity and genotype modulate in situ gene expression of the antarctic bivalve *Aequiyoldia eightsii* in front of a melting glacier

**Supporting Information Figure S1.** CTD profiles of salinity, temperature and turbidity at each station.


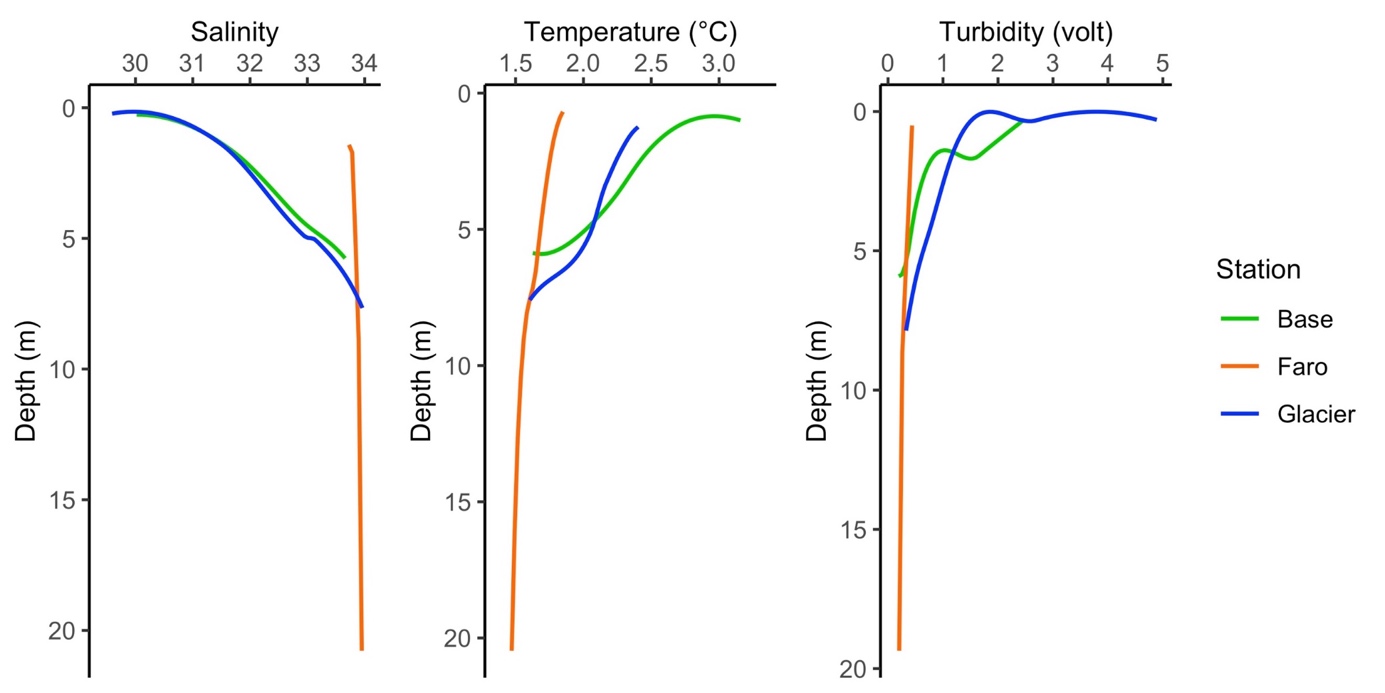


**Supporting Information Table S2.** GO terms, over-represented *p* value and Counts for every mitotype comparison resulting from the GO enrichment analysis based on mitotypes.

| **Category** | **GO term** | **Over-represented *p* value** | **Count** |
| --- | --- | --- | --- |
| **h1h1 vs. h2h2** | | | |
| GO:0005743 | CC mitochondrial inner membrane | 5,39E-06 | 5 |
| GO:0016021 | CC integral component of membrane | 1,22E-05 | 6 |
| GO:0008137 | MF NADH dehydrogenase (ubiquinone) activity | 4,44E-05 | 4 |
| GO:0070469 | CC respiratory chain | 1,02E-04 | 4 |
| GO:0015078 | MF proton transmembrane transporter activity | 2,33E-02 | 1 |
| GO:0015986 | BP ATP synthesis coupled proton transport | 2,33E-02 | 1 |
| GO:0045263 | CC proton-transporting ATP synthase complex | 2,33E-02 | 1 |
| GO:0006119 | BP oxidative phosphorylation | 2,36E-02 | 1 |
| GO:0009060 | BP aerobic respiration | 2,36E-02 | 1 |
| GO:0020037 | MF heme binding | 2,36E-02 | 1 |
| GO:0045277 | CC respiratory chain complex IV | 2,36E-02 | 1 |
| GO:0004129 | MF cytochrome-c oxidase activity | 4,80E-02 | 1 |
| GO:0042773 | BP ATP synthesis coupled electron transport | 4,87E-02 | 1 |
| **h1h3 vs h2h3** | | | |
| GO:0004459 | MF L-lactate dehydrogenase activity | 0,03 | 1 |
| GO:0016887 | MF ATPase activity | 0,03 | 1 |
| GO:0019516 | BP lactate oxidation | 0,03 | 1 |
| GO:0035499 | BP carnosine biosynthetic process | 0,03 | 1 |
| GO:0047730 | MF carnosine synthase activity | 0,03 | 1 |
| GO:0102102 | MF homocarnosine synthase activity | 0,03 | 1 |
| GO:0005743 | CC mitochondrial inner membrane | 0,03 | 3 |
| **h1h1 vs h1h3** | | | |
| GO:0035556 | BP intracellular signal transduction | 0,01 | 9 |
| GO:0016021 | CC integral component of membrane | 0,02 | 19 |
| GO:0003779 | MF actin binding | 0,03 | 5 |
| GO:0004674 | MF protein serine/threonine kinase activity | 0,03 | 7 |
| GO:0006468 | BP protein phosphorylation | 0,03 | 7 |
| GO:0070469 | CC respiratory chain | 0,03 | 9 |
| GO:0046872 | MF metal ion binding | 0,03 | 11 |
| **h1h1 vs h2h3** | | | |
| GO:0016021 | CC integral component of membrane | 1,96E-06 | 18 |
| GO:0005743 | CC mitochondrial inner membrane | 3,37E-05 | 10 |
| GO:0070469 | CC respiratory chain | 1,05E-03 | 8 |
| GO:0008137 | MF NADH dehydrogenase (ubiquinone) activity | 1,48E-03 | 7 |
| GO:0006468 | BP protein phosphorylation | 1,65E-03 | 6 |
| GO:0004674 | MF protein serine/threonine kinase activity | 1,68E-03 | 6 |
| GO:0007275 | BP multicellular organism development | 6,35E-03 | 5 |
| GO:0005737 | CC cytoplasm | 7,57E-03 | 15 |
| GO:0000287 | MF magnesium ion binding | 8,73E-03 | 4 |
| GO:0035556 | BP intracellular signal transduction | 8,78E-03 | 6 |
| GO:0005886 | CC plasma membrane | 1,57E-02 | 6 |
| GO:0005576 | CC extracellular region | 2,25E-02 | 4 |
| GO:0046872 | MF metal ion binding | 2,59E-02 | 7 |
| GO:0001669 | CC acrosomal vesicle | 3,34E-02 | 3 |
| GO:0004888 | MF transmembrane signaling receptor activity | 4,51E-02 | 2 |
| GO:0051260 | BP protein homooligomerization | 4,56E-02 | 2 |
| GO:0005525 | MF GTP binding | 4,56E-02 | 4 |
| GO:0007017 | BP microtubule-based process | 4,56E-02 | 4 |
| GO:0035036 | BP sperm-egg recognition | 4,61E-02 | 2 |
| GO:0060388 | CC vitelline envelope | 4,61E-02 | 2 |
| GO:0044877 | MF protein-containing complex binding | 4,65E-02 | 2 |
| GO:0042773 | BP ATP synthesis coupled electron transport | 4,86E-02 | 2 |
| **h2h2 vs h2h3** | | | |
| GO:0005737 | CC cytoplasm | 9,13E-06 | 28 |
| GO:0007275 | BP multicellular organism development | 1,38E-03 | 7 |
| GO:0035556 | BP intracellular signal transduction | 1,63E-03 | 9 |
| GO:0005874 | CC microtubule | 1,11E-02 | 8 |
| GO:0004674 | MF protein serine/threonine kinase activity | 1,19E-02 | 7 |
| GO:0006468 | BP protein phosphorylation | 1,30E-02 | 7 |
| GO:0001669 | CC acrosomal vesicle | 2,63E-02 | 4 |
| GO:0005634 | CC nucleus | 3,26E-02 | 12 |
| GO:0007286 | BP spermatid development | 4,60E-02 | 8 |

**Supporting Information Table S3.** Examples of differentially expressed genes associated with the top three most significant GO terms for each mitotype comparison

| **Transcript ID** | **Annotation** |
| --- | --- |
|  |  |
| **h1h1 vs h1h3** | |
| *GO:0035556: intracellular signal transduction* | |
| TRINITY_DN368515_c0_g1 | Testis-specific serine/threonine-protein kinase |
| TRINITY_DN24821_c0_g1 | Testis-specific serine/threonine-protein kinase 1 |
| TRINITY_DN25668_c0_g1 | Testis-specific serine/threonine-protein kinase 1 |
| *GO:0016021: integral component of membrane* | |
| TRINITY_DN363456_c1_g1 | NADH-ubiquinone oxidoreductase chain 4L |
| TRINITY_DN368531_c0_g1 | NADH-ubiquinone oxidoreductase chain 5 |
| TRINITY_DN335502_c0_g1 | NADH-ubiquinone oxidoreductase chain 1 |
| GO:0003779: actin binding | |
| TRINITY_DN27357_c0_g1 | Vacuolar protein sorting-associated protein |
| TRINITY_DN80715_c3_g1 | Unconventional myosin-IXAb |
| TRINITY_DN39169_c0_g1 | Kelch-like protein 1 |
|  |  |
| **h1h1 vs h2h2** | |
| *GO:0005743: mitochondrial inner membrane* | |
| TRINITY_DN6_c2_g2 | Cytochrome c oxidase subunit 1 |
| TRINITY_DN75_c5_g1 | NADH-ubiquinone oxidoreductase chain 1 |
| TRINITY_DN7902_c1_g1 | NADH-ubiquinone oxidoreductase chain 5 |
| *GO:0016021: integral component of membrane* | |
| TRINITY_DN6_c2_g2 | Cytochrome c oxidase subunit 1 |
| TRINITY_DN7902_c0_g1 | NADH-ubiquinone oxidoreductase chain 5 |
| TRINITY_DN7664_c2_g1 | NADH-ubiquinone oxidoreductase chain 4 |
| *GO:0008137: NADH dehydrogenase (ubiquinone) activity* | |
| TRINITY_DN75_c5_g1 | NADH-ubiquinone oxidoreductase chain 1 |
| TRINITY_DN7902_c1_g1 | NADH-ubiquinone oxidoreductase chain 5 |
| TRINITY_DN7902_c0_g1 | NADH-ubiquinone oxidoreductase chain 5 |
|  |  |
| **h1h1 vs h2h3** | |
| *GO:0016021: integral component of membrane* | |
| TRINITY_DN363456_c1_g1 | NADH-ubiquinone oxidoreductase chain 4L |
| TRINITY_DN368531_c0_g1 | NADH-ubiquinone oxidoreductase chain 5 |
| TRINITY_DN335502_c0_g1 | NADH-ubiquinone oxidoreductase chain 1 |
| *GO:0005743: mitochondrial inner membrane* | |
| TRINITY_DN1947_c0_g1 | NADH-ubiquinone oxidoreductase chain 1 |
| TRINITY_DN8743_c0_g1 | Cytochrome b |
| TRINITY_DN6_c2_g2 | Cytochrome c oxidase subunit 1 |
| *GO:0070469: respiratory chain* | |
| TRINITY_DN75_c5_g1 | NADH-ubiquinone oxidoreductase chain 1 |
| TRINITY_DN615_c0_g1 | Cytochrome b |
| TRINITY_DN335502_c0_g1 | NADH-ubiquinone oxidoreductase chain 1 |
|  |  |
| **h1h3 vs h2h3** | |
| *GO:0004459: L-lactate dehydrogenase activity* | |
| TRINITY_DN5064_c4_g1 | Carnosine synthase 1 |
| *GO:0005743: mitochondrial inner membrane* | |
| TRINITY_DN7902_c0_g1 | NADH-ubiquinone oxidoreductase chain 5 |
| *GO:0019516: lactate oxidation* | |
| TRINITY_DN5064_c4_g1 | Carnosine synthase 1 |
|  |  |
| **h2h2 vs h2h3** | |
| *GO:0005737: cytoplasm* | |
| TRINITY_DN368515_c0_g1 | Testis-specific serine/threonine-protein kinase 5 |
| TRINITY_DN363338_c0_g1 | Tubulin beta-4B chain |
| TRINITY_DN24821_c0_g1 | Serine/threonine-protein kinase 1 |
| *GO:0007275: multicellular organism development* | |
| TRINITY_DN58702_c0_g1 | Testis-specific serine/threonine-protein kinase 4 |
| TRINITY_DN5838_c0_g1 | Testis-specific serine/threonine-protein kinase 3 |
| TRINITY_DN15948_c2_g2 | Testis-specific serine/threonine-protein kinase 2 |
| *GO:0035556: intracellular signal transduction* | |
| TRINITY_DN25668_c0_g1 | Testis-specific serine/threonine-protein kinase 1 |
| TRINITY_DN368515_c0_g1 | Testis-specific serine/threonine-protein kinase 5 |
| TRINITY_DN62156_c0_g1 | Calcium-dependent protein kinase 24 |

**Supporting Information Table S4.** GO terms and associated Biological processes for single differential expressed genes resulting from differential expression analysis (DEA) by stations and nuclear genotype.

| DEA by stations – biological processes | |
| --- | --- |
| Base vs. Faro | |
| Term ID | Description |
| GO:0007155 | cell adhesion |
| GO:0009609 | response to symbiotic bacterium |
| GO:0042391 | regulation of membrane potential |
| GO:0050877 | neurological system process |
| GO:0051260 | protein homooligomerization |
| GO:0070715 | sodium-dependent organic cation transport |
| GO:0006637 | acyl-CoA metabolic process |
| GO:0006006 | glucose metabolic process |
| GO:0070936 | protein K48-linked ubiquitination |
| GO:0007268 | chemical synaptic transmission |
| GO:0009650 | UV protection |
| GO:0035499 | carnosine biosynthetic process |
| GO:0010506 | regulation of autophagy |
| GO:0055114 | oxidation-reduction process |
| GO:0051726 | regulation of cell cycle |
| GO:0043161 | proteasome-mediated ubiquitin-dependent protein catabolic process |
| GO:0019516 | lactate oxidation |
| GO:0042981 | regulation of apoptotic process |
| GO:1990961 | drug transmembrane export |
| GO:0015697 | quaternary ammonium group transport |
| GO:0006544 | glycine metabolic process |
| GO:0071447 | cellular response to hydroperoxide |
| GO:0097340 | inhibition of cysteine-type endopeptidase activity |
| GO:0006814 | sodium ion transport |
| GO:0006468 | protein phosphorylation |
| GO:0046474 | glycerophospholipid biosynthetic process |
| GO:0097435 | supramolecular fiber organization |
| GO:0048251 | elastic fiber assembly |
|  |  |
| Base vs. Glacier | |
| Term ID | Description |
| GO:0006979 | response to oxidative stress |
| GO:0007155 | cell adhesion |
| GO:0019835 | cytolysis |
| GO:0070177 | contractile vacuole discharge |
| GO:0033298 | contractile vacuole organization |
| GO:0070936 | protein K48-linked ubiquitination |
| GO:0048800 | antennal morphogenesis |
| GO:0042438 | melanin biosynthetic process |
| GO:2001225 | regulation of chloride transport |
| GO:0000281 | mitotic cytokinesis |
| GO:0032849 | positive regulation of cellular pH reduction |
| GO:0006915 | apoptotic process |
| GO:0017148 | negative regulation of translation |
| GO:0000463 | maturation of LSU-rRNA from tricistronic rRNA transcript (SSU-rRNA, 5.8S rRNA, LSU-rRNA) |
| GO:0009650 | UV protection |
| GO:0033146 | regulation of intracellular estrogen receptor signaling pathway |
| GO:0031038 | myosin II filament organization |
| GO:0048251 | elastic fiber assembly |
| GO:0031288 | sorocarp morphogenesis |
| GO:0010712 | regulation of collagen metabolic process |
| GO:0006468 | protein phosphorylation |
|  |  |
| Glacier vs. Faro | |
| Term ID | Description |
| GO:0043627 | response to estrogen |
| GO:0061614 | pri-miRNA transcription from RNA polymerase II promoter |
| GO:0005975 | carbohydrate metabolic process |
| GO:1904184 | positive regulation of pyruvate dehydrogenase activity |
| GO:0010288 | response to lead ion |
| GO:0001666 | response to hypoxia |
| GO:0006412 | translation |
| GO:0042594 | response to starvation |
| GO:0051412 | response to corticosterone |
| GO:0010033 | response to organic substance |

| DEA by nuclear genotype – biological processes | |
| --- | --- |
|  |  |
| NucA vs NucB | |
| Term ID | Description |
| GO:0009609 | response to symbiotic bacterium |
| GO:0010506 | regulation of autophagy |
| GO:0030214 | hyaluronan catabolic process |
| GO:0048800 | antennal morphogenesis |
| GO:0070715 | sodium-dependent organic cation transport |
| GO:0000463 | maturation of LSU-rRNA from tricistronic rRNA transcript |
| GO:0070936 | protein K48-linked ubiquitination |
| GO:0071447 | cellular response to hydroperoxide |
| GO:0046653 | tetrahydrofolate metabolic process |
| GO:0097340 | inhibition of cysteine-type endopeptidase activity |
| GO:0055114 | oxidation-reduction process |
| GO:0051726 | regulation of cell cycle |
| GO:0016055 | Wnt signaling pathway |
| GO:1990961 | drug transmembrane export |
| GO:0015697 | quaternary ammonium group transport |
| GO:0060731 | positive regulation of intestinal epithelial structure maintenance |
| GO:0006814 | sodium ion transport |
| GO:0033514 | L-lysine catabolic process to acetyl-CoA via L-pipecolate |
| GO:0009166 | nucleotide catabolic process |
| GO:0006468 | protein phosphorylation |
